# Early Post‐Release Movement Convergence in Reintroduced Giant Pandas

**DOI:** 10.1002/ece3.73807

**Published:** 2026-06-09

**Authors:** Yuxin Jiang, Shengshan He, Tianjiao Liu, Yuhang Li, Yuanhao Du, Xiaoqiang Liu, Qian Wang, Haohong Wan, Desheng Li, Jianghong Ran

**Affiliations:** ^1^ Sichuan Key Laboratory of Conservation Biology on Endangered Wildlife Sichuan University Chengdu Sichuan China; ^2^ The Conservation of Endangered Wildlife Key Laboratory of Sichuan Province Chengdu Sichuan China; ^3^ Key Laboratory of SFGA on the Giant Panda China Conservation and Research Center for the Giant Panda Chengdu Sichuan China; ^4^ Laboratory of Animal Behavior and Conservation, School of Life Sciences Nanjing University Nanjing Jiangsu China

**Keywords:** behavioral plasticity, giant panda, movement ecology, spatial behavior

## Abstract

Energy budgets are a fundamental constraint on animal movement, shaping both spatial use and behavioral strategies. When energy is plentiful and risks are low, animals often benefit by investing more in movement. Yet, how movement patterns shift under extreme energetic limitations remains poorly understood. In this study, we used the giant panda ( *Ailuropoda melanoleuca* ), a species with pronounced energetic constraints, as a model to examine movement adaptations under the dual challenges of dietary specialization and very low energy utilization efficiency. We tracked eight reintroduced pandas with global positioning system (GPS) collars over 474 days. Principal component analysis of 10 movement parameters revealed two independent behavioral axes: an Exploration–Exploitation axis (51.48% of variance) and a Tortuosity‐Consistency axis (21.25% of variance). Both axes showed significant initial repeatability (*R* = 0.16–0.26) that diminished over time and stabilized near 0.2. Notably, initially exploratory individuals gradually reduced their space‐use range, while exploitative individuals maintained more consistent space use, leading to decreased among‐individual variation during the early acclimation period after release. This pattern aligns with the panda's bamboo diet and low energy expenditure reported in previous studies. Building on this, we propose a conceptual “Diet‐Energy Utilization Constraint” hypothesis to guide future research into how dietary specialization and energy efficiency influence movement adaptation post‐release.

## Graphical abstract

We used high‐frequency GPS tracking of eight reintroduced giant pandas (474 tracking days) to test how extreme energetic constraints shape movement during early post‐release adjustment. Principal component analysis of ten daily movement metrics revealed two independent movement axes (exploration–exploitation and tortuosity–consistency), and an expanding‐window repeatability analysis showed that among‐individual differences declined over time, indicating convergence toward energy‐saving strategies. These findings support a conceptual Diet–Energy Utilization Constraint framework linking diet and net energy gain to movement flexibility and behavioral convergence.

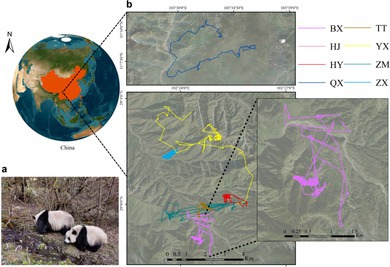

## 1 Introduction

Accurately predicting how animals move in response to environmental change is vital for the effective conservation of species worldwide (Gomez et al. 2025; Wrensford et al. 2025). Movement is recognized as a key behavioral response to environmental shifts (Riotte‐Lambert and Matthiopoulos 2020) and is manifested through activities such as migration, dispersal, foraging, and exploration (Bowler and Benton 2005; Cooke et al. 2024; Gauzens et al. 2024). Interindividual variation in movement, driven by experience and additive genetic effects (Brønnvik and Flack 2025; Cockrem 2013; Dochtermann et al. 2015), has been shown to facilitate population adaptation to changing environments (Forsman and Wennersten 2016). However, this variation is not fixed, and adaptive costs, particularly increased energy expenditure, are typically incurred by individuals (Murren et al. 2015). Because energy budgets are limited, individuals may face trade‐offs between movement and other physiological demands, ultimately limiting movement dynamics (Boratyński 2020; Bright Ross et al. 2024; Zhao and Cao 2009). For example, in the humpback whale ( *Megaptera novaeangliae* ), females reduce movement‐related energy expenditure through resting behavior during the breeding season, allowing additional energy to be allocated to reproduction (Bejder et al. 2019). Therefore, a comprehensive understanding of how energy budgets constrain movement is essential for the accurate prediction and effective management of vulnerable species under environmental change (Fox et al. 2019; Moritz and Agudo 2013; Wong and Candolin 2014).

Even under identical environmental conditions, individuals within the same population often exhibit divergent energy budgets and movement strategies as a result of variation in body condition (Cecere et al. 2020; Grémillet et al. 2018; Hertel et al. 2020; Lyu et al. 2021). This variation influences not only the physiological determinants of energy requirements—such as life‐history traits and allometric scaling (Bright Ross et al. 2024)‐but is also expressed behaviorally; bolder and faster exploratory movements are consistently exhibited by certain individuals (Dingemanse et al. 2010). Such consistent interindividual behavioral differences across time and contexts are referred to as animal personality or behavioral types (BTs) (Bar‐Ziv et al. 2025; Sih et al. 2004). Along the proactive‐reactive axis, greater boldness and aggressiveness are typically exhibited by proactive individuals, who explore new environments rapidly and superficially, whereas the opposite tendencies are characteristic of reactive individuals (Koolhaas et al. 1999). However, in variable environments, greater movement plasticity is often exhibited by reactive individuals than by proactive individuals, allowing them to adapt more readily to novel conditions (Cockrem 2007, 2022; Dingemanse and Réale 2005). For instance, in Atlantic cod ( *Gadus morhua* ), rising sea temperatures are met by reactive individuals, who reduce home‐range size, reflecting their lower energy demands (Villegas‐Ríos et al. 2018). Despite these insights, empirical investigations of the relationship between energy budgets and movement have predominantly been conducted under conditions of relatively abundant energy supply. For example, in dingoes ( *Canis dingo* ), significant increases in movement intensity have been documented under conditions of abundant food resources (Tatler et al. 2021). By contrast, the effects of severe energy constraints on movement, particularly how individuals of different behavioral types adjust their strategies under such limitations, have received little empirical attention.

Movement is fundamentally governed by a trade‐off between energy intake and expenditure (Chimienti et al. 2020). This trade‐off is constrained by digestive physiology; gut length, digestive enzyme activity, and gut microbiota collectively determine the efficiency with which energy is extracted from food (Karasov and Douglas 2013). In dietary specialists, digestive specialization is thought to enhance the processing efficiency of specific foods while imposing an upper limit on total energy intake. For instance, in herbivores, longer digestion times and larger gut capacities are required, directly constraining the energy available for movement (Van Soest 1994). In contrast, diverse food resources can be utilized by generalist individuals to maintain energy intake (Mori et al. 2019). Therefore, an individual's total energy income is jointly determined by dietary breadth, energy utilization efficiency, and resource availability in its particular home range over space and time (De Cuyper et al. 2020; Hefty and Stewart 2019; Mitchell and Powell 2004; Peyré‐Tartaruga and Coertjens 2018; Richard et al. 2017). Significant differences in energy demands for movement are exhibited by individuals of different behavioral types; the fast, wide‐ranging exploration spatial pattern characteristic of proactive individuals necessitates higher movement‐related energy expenditure (Careau et al. 2008). Previous research has demonstrated that proactive common brushtail possums (*Trichosurus vulpecula*), as herbivores, support their high‐energy movement patterns by adopting a more diverse diet and selecting higher‐quality food (Herath et al. 2021). However, when dietary breadth is narrow and energy utilization efficiency is low, movement plasticity patterns can be shaped by energy budgets in ways that depart from conventional expectations across behavioral types. In variable environments, proactive individuals may display greater movement plasticity under energetic pressure, whereas reactive individuals, whose movements require less energy, may possess a more restricted capacity for plasticity. Empirical evidence regarding the effects of energy budgets on animal movement plasticity remains limited, with existing studies (Dantzer et al. 2020; Studd et al. 2020) focusing primarily on the locomotor regulation of small animals under energetic restriction. By contrast, investigations of the interplay between movement plasticity and energetic constraints in large animals have been largely confined to theoretical frameworks, owing to methodological difficulties, high costs, and ethical considerations. Accordingly, it remains unclear how movement plasticity varies among behavioral types in large animals facing extreme energetic constraints. It is also unknown whether the expected plasticity hierarchy between proactive and reactive phenotypes may even be inverted.

The giant panda (*Ailuropoda melanoleuca*) is widely regarded as a dietary specialist with extremely energetic constraints among mammals. As an obligate bamboo feeder, the giant panda has a daily energy expenditure that is only 38% of the expected value for a mammal of its size, making it one of the largest mammals with the lowest metabolic rates (Nie et al. 2015). The giant panda reintroduction program is considered a valuable “natural experiment” for studying movement adaptation under extreme energy constraints. After release, all individuals entered broadly similar habitats with sufficient foraging resources and had to develop new space‐use patterns in unfamiliar landscapes. Global positioning system (GPS) tracking recorded their post‐release adjustment process in detail. Consequently, a unique opportunity is provided to examine the impact of extreme energy constraints on movement patterns during the early post‐release period in a novel environment.

Eight reintroduced subadult giant pandas were tracked, yielding continuous movement trajectory data totaling 474 days. The objectives of this study are as follows: (i) to quantify the temporal dynamics and individual consistency of giant‐panda movement patterns and to ask whether among‐individual differences weakened during the early post‐release period; (ii) to analyze differences among individuals in baseline behavioral levels and the magnitudes of behavioral change, thereby characterizing the adaptation process of a dietary specialist in a novel environment, specifically, how initially exploratory individuals gradually contract their activity ranges and how individual variation shapes this short‐term adjustment process; and (iii) to propose a conceptual Diet–Energy Utilization hypothesis, based on the observed movement patterns in giant pandas, that may guide future tests of how dietary specialization and energy utilization efficiency relate to movement adaptation after release. Given the giant panda's specialized bamboo diet and exceptionally low reported daily energy expenditure, we hypothesized that sustained wide‐ranging exploration would be difficult to maintain during the early post‐release period. Accordingly, we predicted that movement would show detectable among‐individual variation shortly after release, reflecting differences in initial exploratory tendencies. As individuals accumulated local information in unfamiliar landscapes, initially, more exploratory individuals may gradually reduce movement extent, leading to a weakening of among‐individual differences over time.

## 2 Method

### 2.1 Study Area and GPS Tracking

The study was conducted in the Liziping Nature Reserve (LNR) and the Longxi‐Hongkou National Nature Reserve (LHNNR) in Sichuan Province, China. Both reserves contain extensive bamboo‐dominated understory typical of giant panda habitat, providing the primary food resource for released individuals. From 2012 to 2018, eight subadult giant pandas released into the wild were fitted with GPS collars (Lotek Engineering, Newmarket, Ontario, Canada), with a sampling frequency of every 2 h (including latitude, longitude, and elevation). The 2‐h interval was determined by the GPS‐collar settings used during post‐release monitoring. A similar few‐hour GPS interval has been used in a previous giant panda movement study, including 4‐h fixes for quantifying activity patterns in wild giant pandas (Zhang et al. 2015). Although releases occurred across multiple years (2012–2018), our analyses used only the first 1–2 months of GPS data after each individual's release. This restriction reflected our focus on early post‐release spatial adjustment. Previous GPS‐based work on reintroduced giant pandas showed that captive‐bred individuals required approximately 3–5 months to complete the initial adjustment process after release (He et al. 2019). Therefore, the short‐term tracking window used here captured the initial search and settlement phase before long‐term movement patterns were expected to be fully established. Tracking duration ranged from 32 to 65 days per individual (Table 1), with a mean duration of 59.25 ± 11.07 days, yielding 474 tracking days in total across all individuals. According to recent post‐release monitoring records from the China Conservation and Research Center for the Giant Panda (CCRCGP), all eight individuals included in our study were successful release cases. All GPS fixes used in the analyses were collected while the collars were functioning and the individuals were under active post‐release monitoring. Although movement immediately after translocation may include unusual responses associated with unfamiliarity with the new environment, we did not censor an initial acclimation period because our aim was to characterize the full early post‐release adjustment process from the time of release onward.

**TABLE 1:** Details of the release of giant pandas.

ID	Sex	Age	Tracking days	Release site
HY	Female	2	32	LNR
ZX	Female	2	61	LNR
ZM	Female	3	65	LNR
YX	Female	2	63	LNR
BX	Male	2	63	LNR
HJ	Female	2	63	LNR
TT	Male	2	63	LNR
QX	Female	2	64	LHNNR

**FIGURE 1:**
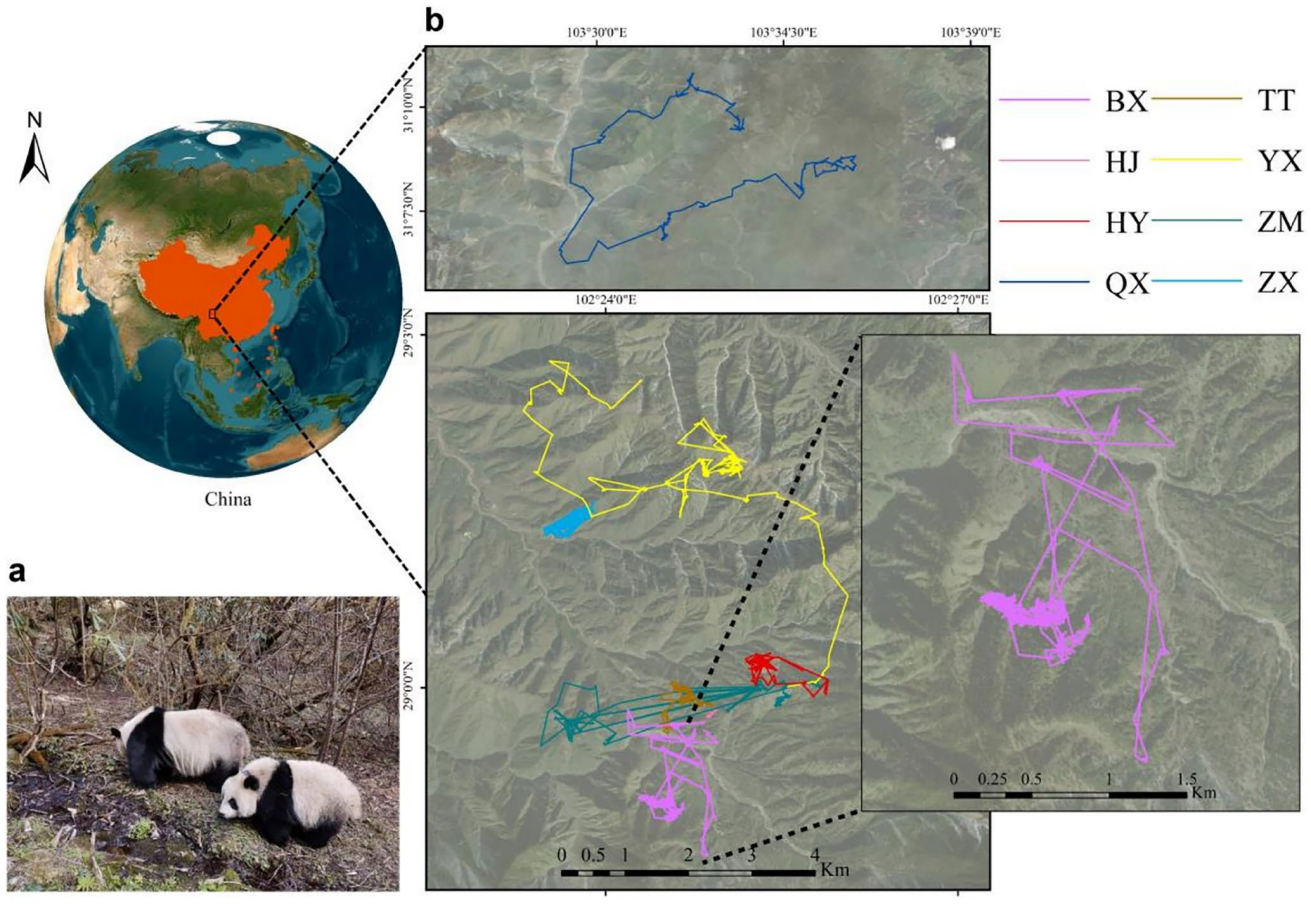
(a) Giant panda ( *Ailuropoda melanoleuca* ), equipped with a GPS collar. (b) Trajectories of released individuals over 2 months. Each color represents a different giant panda individual.

The eight subadult giant pandas tracked in this study were all captive‐bred. The pandas were raised in captivity to enhance their survival skills in the wild. We collected the movement tracks for the first 2 months after release for all eight individuals (six females and two males; Figure 1). Due to GPS data loss for individual “HY” in the second month, only the first month's movement track was retained (Table 1). We chose not to interpolate missing fixes, as doing so would introduce model‐derived locations into the movement paths and potentially distort daily path metrics, particularly those involving distance, turning angles, straightness, and revisit patterns. Consequently, we based all movement metrics solely on observed fixes to ensure they reflected actual recorded positions rather than inferred trajectories. While this approach may have reduced temporal resolution on days with missing fixes and resulted in more conservative estimates for certain movement metrics, it prevented the introduction of interpolation‐related bias into daily summaries.

### 2.2 Movement Data Analysis

Before calculating movement metrics, we performed basic quality control on the GPS data by removing duplicate records, excluding erroneous locations, and discarding records with missing latitude or longitude values.

We characterized daily movements using several movement indices derived from their trajectories. Additionally, we divided the study area into longitude and latitude grids with a resolution of 0.001 degrees to compute the repeat ratio. Overall, we calculated 10 movement indices: (1) daily total distance (in meters); (2) mean turning angle (degree); (3) straightness index (calculated by comparing the ratio of the total distance to the net displacement); (4) mean speed (meters per second); (5) repeat ratio; (6) max diameter (the distance between the two most distant locations in a daily trajectory, in meters); (7) revisit rate; (8) direction consistency; (9) mean elevation change (meters per 2 h); and (10) mean nearest neighboring distance (in meters).

The ten daily movement metrics were designed to capture complementary aspects of intensity, spatial extent, and path geometry. Metrics such as daily total distance, mean speed, and maximum diameter describe movement investment and spatial extent; straightness and turning angle reflect path tortuosity; repeat ratio and revisit rate quantify within‐day reuse of space (indicative of localized exploitation versus exploration); and direction consistency describes how persistently an individual maintains a movement bearing over successive steps (values closer to 1 indicate more consistent direction).

### 2.3 Statistical Analysis

All analyses were conducted in R v. 4.4.2 and RStudio. After extracting the 10 movement indices described above, we assessed temporal and spatial autocorrelation to understand the structure of dependencies in our data. For temporal autocorrelation, we calculated the autocorrelation function (ACF) at lag‐1 for each individual and performed Ljung‐Box tests. According to movement metrics, which were summarized at a daily resolution, our analyses focused on short‐term post‐release change, we used lag‐1 ACF and Ljung‐Box tests as pragmatic diagnostics of residual temporal dependence at the timescale most relevant to early adjustment. For spatial autocorrelation, we computed Moran's *I* using the ape package based on pairwise distances between daily location centroids. Indices were classified as having significant temporal autocorrelation if |ACF| > 0.3 or if the Ljung‐Box test produced *p* < 0.05, and significant spatial autocorrelation if Moran's *I* test produced *p* < 0.05. We retained the temporal structure in our data, as these temporal changes were biologically meaningful and captured the early post‐release adjustment process that we sought to quantify. Meanwhile, to control spatial autocorrelation arising from spatial clustering of activities, we calculated daily spatial centroids (mean longitude and latitude) for each individual. For movement indices with significant spatial autocorrelation, these spatial coordinates were included as covariates in subsequent models. This covariate‐based approach aimed to address broad‐scale spatial clustering efficiently, while remaining suitable for our limited sample size and brief observation period. For indices without significant spatial autocorrelation, we applied no spatial correction. This approach does not account for higher‐order temporal dependence or more complex nonlinear spatial patterns, and should thus be regarded as a practical correction rather than a comprehensive spatiotemporal error model.

We conducted analyses of the trend of movement parameters using linear mixed‐effects models (LMMs) (Nakagawa and Schielzeth 2010). We constructed separate linear mixed models (LMMs) for each of the 10 movement indices, using day since release as a fixed effect and individual identity as a random intercept. For indices showing significant spatial autocorrelation, daily centroid longitude and latitude were included as additional fixed covariates to account for broad‐scale spatial clustering. Then, to assess model explanatory power, conditional *R* ^2^ values were calculated using the package “MuMIn.” Furthermore, to determine the consistency of individual movement, we calculated repeatability for each activity index using the “*rptR*” package. Movement indices with significant repeatability (*p* < 0.05) were included in the PCA via the “prcomp” package from the “stat” package in R. In addition, we first transformed them to fit a normal distribution, applied a log transformation to each movement index (log (*x* + 1)) to approximate normality, and then modeled each index with individual identity as a random effect and 1000 bootstraps for confidence interval estimation, using the package “rptR.” Then, we estimated repeatability for PC1 and PC2 and examined its temporal dynamics using an expanding‐window (day ≤ *d*) cumulative repeatability approach implemented in *rptR* (Gaussian models, 1000 bootstraps), with day as a fixed effect and ID as a random intercept. For each axis, we fitted two LMMs with day since release as a fixed effect and individual identity as a random effect. The first model allowed individuals to differ only in baseline values, whereas the second also allowed them to differ in the rate of change over time. We compared the two models using likelihood‐ratio tests to determine whether including individual‐specific temporal slopes improved model fit. Then, we extracted the variances of the random intercepts and random slopes, along with their 95% confidence intervals, from the conditional posterior variances.

## 3 Result

Eight giant pandas were included in our analysis. Overall, our results indicate that individual giant pandas displayed consistent differences in movement patterns during the early post‐release phase in a novel environment. Each individual adopted distinct movement strategies throughout this short‐term adjustment period, as described below.

### 3.1 Temporal Dynamics and Individual Variation of Movement Indices in Giant Pandas

To investigate temporal trends in giant panda movement, we used linear mixed‐effects models to assess time‐related changes in 10 movement indices. The results showed that the trends in each movement index over time were insignificant (*p* > 0.05; Figure S1). These results indicate that the raw movement indices did not change in a single shared direction across all individuals after release. Individual pandas showed heterogeneous temporal trajectories, with movement indices changing in different directions and at different rates during early post‐release adjustment.

### 3.2 Giant Pandas Exhibit Different Spatial Behavior Types in Novel Environments

**FIGURE 2:**
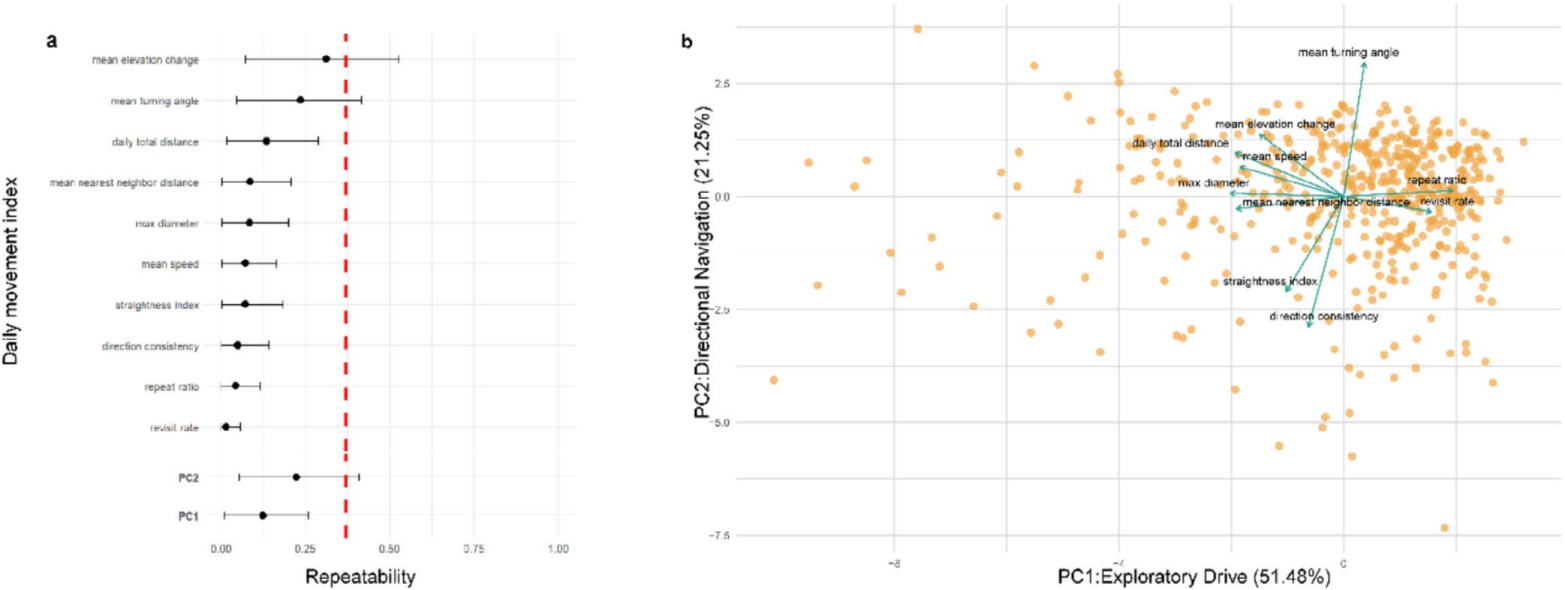
Repeatability and principal component analysis (PCA) of movement indices summarizing 474 days of 8 giant pandas. (a) Repeatability values and their 95% confidence intervals for movement indices are included in the PCA. The repeatability of PC1 and PC2 is also presented at the bottom. The vertical red‐dashed line indicates that *R* = 0.37 is suggested as the mean of BT (Bell et al. 2009). (b) A PCA bi‐plot showing daily values and loading for the ten indices of daily movements PC1 (51.48% variance explained): Exploration–exploitation (E×E) and PC2 (21.25% variance explained): Tortuosity‐consistency (T×C). Points indicate specific days. Higher PC values for the two axes indicate a shorter distance of movement, reuse of resources from the same location, and frequent turns.

To assess whether giant pandas exhibit repeatable spatial behavioral types, we evaluated the repeatability of 10 daily movement indices and two principal components (PCs) derived from principal component analysis (PCA). All 10 indices showed statistically supported repeatability (*p* < 0.05; Figure 2a), indicating detectable among‐individual differences in these movement metrics. The repeatability magnitudes varied across indices (see *R* estimates and 95% CIs in Figure 2a). We then selected the repeatable indices for dimensionality reduction via PCA. The two PCs explained 51.48% and 21.25% of the total variance, within eigenvalues of 5.15 and 2.13, for PC1 and PC2, respectively (Figure 2b). We extracted the variables with an absolute value of the load > 0.3 and named PC1 and PC2 based on the ecological significance of the variable loads (Table S1). We interpreted PC1 as representing *Exploitation–Exploration* (negative loading: daily total distance; mean speed; daily max diameter; mean elevation change; mean near‐nearest distance; positive loading: repeat ratio; revisit rate; E×E). Lower PC1 scores reflect movement that covers a wider area at a quicker pace and involves more exploration. In contrast, higher PC1 scores correspond to movement that is concentrated in a smaller region and follows more repetitive patterns. PC2 was primarily associated with variables linked to the direction of movement, including straightness index, turning angles, and directional consistency. We interpreted PC2 as representing *Tortuosity‐Consistency* (negative loading: straightness and directional consistency; positive loading: turning angle; T×C). Higher PC2 scores reflect movement that is more winding and shows less consistent directionality. Both PCs demonstrated moderate within‐individual repeatability across time windows (PC1: *R* = 0.16, 95% CI = [0.04, 0.31], *p* < 0.05; PC2: *R* = 0.26, 95% CI = [0.06, 0.46], *p* < 0.05). Moreover, the two axes were statistically independent and uncorrelated at the individual level (*ρ* = −0.004, *p* = 0.99).

### 3.3 Temporal Dynamics of Spatial Behavior Types of Repeatability

**FIGURE 3:**
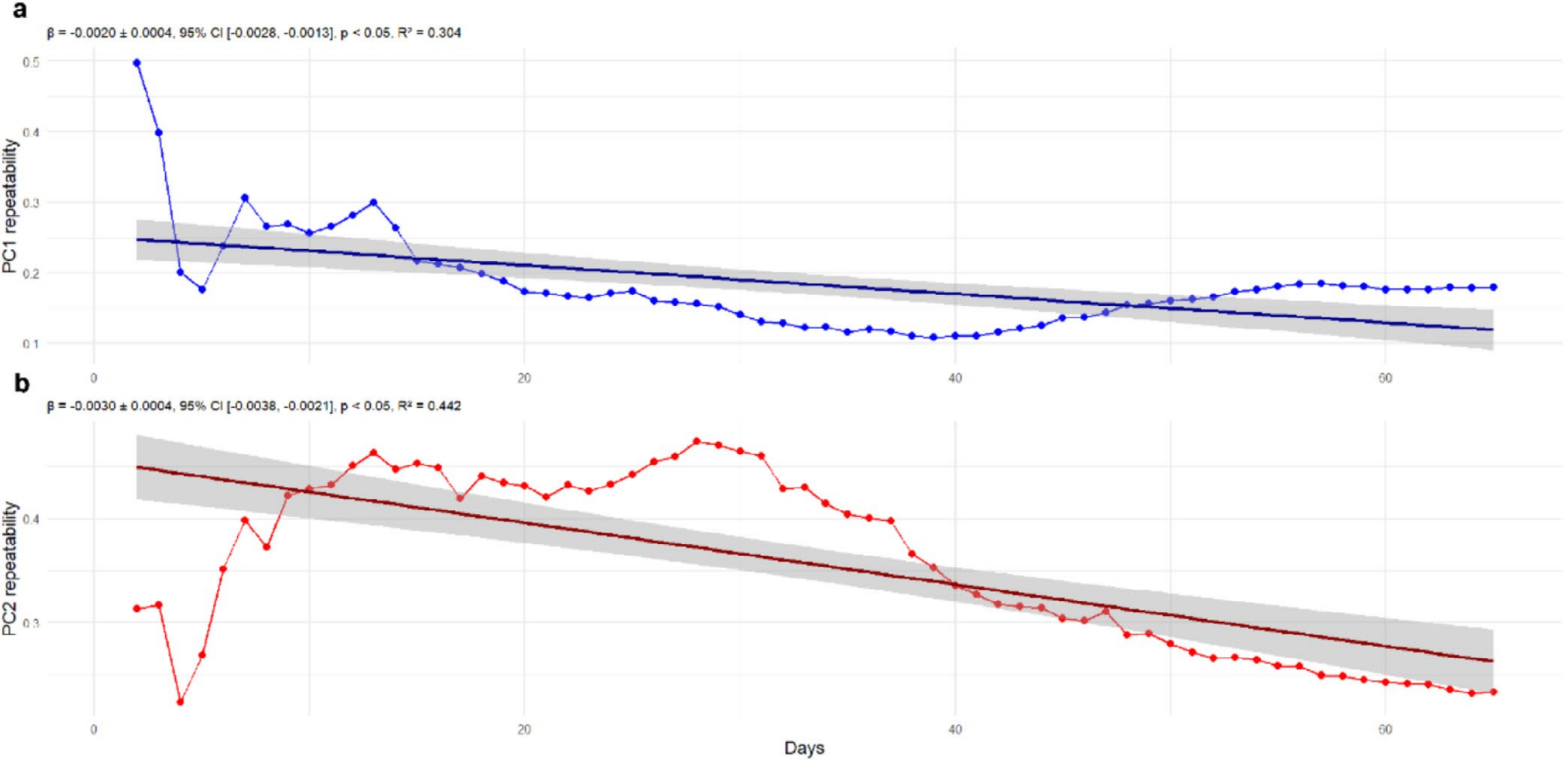
The cumulative repetitiveness of two types of spatial behaviors over time. The points represent the repeatability value for a given day, and the solid line represents the regression curve along with the 95% confidence interval. (a) The trend of cumulative repetitiveness for PC1 over time. (b) The trend of cumulative repetitiveness for PC2 over time.

Having identified two independent spatial behavior types in giant pandas, we next examined their temporal dynamics using linear mixed‐effects models and linear models. Both E×E and T×C exhibited significant repeatability in a novel environment (PC1: β = −0.002 ± 0.0004, 95% CI = [−0.0028, −0.0013], *p* < 0.01, *R* ^2^ = 0.304; PC2: β = −0.003 ± 0.0004, 95% CI = [−0.0038, −0.0021], *p* < 0.01, *R* ^2^ = 0.442), with a consistent decline in repeatability over time (Figure 3). Eventually, repeatability for both components stabilized around 0.2.

### 3.4 Different Movement Strategies of Giant Pandas

**FIGURE 4:**
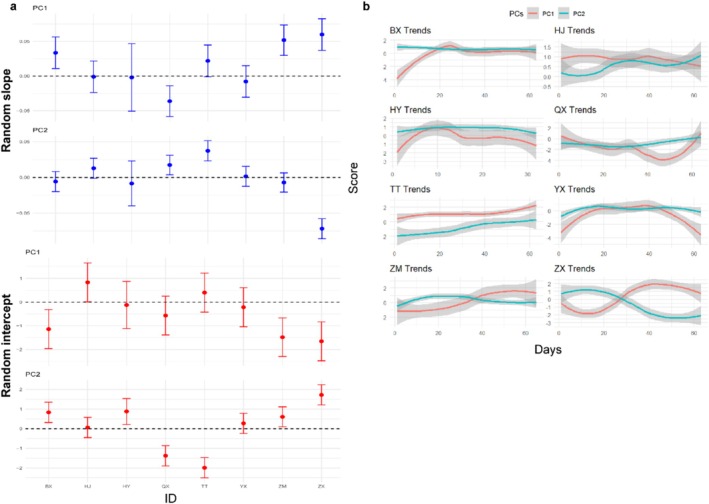
The analysis of random effects for two types of spatial behaviors and the temporal changes of individual trajectories in PC1 and PC2 are shown in the figure. (a) The figure presents the random slope estimates and their confidence intervals for PC1 and PC2, with blue dots representing the random slope estimates for everyone and blue error bars indicating the 95% confidence intervals of these estimates. The black dashed line represents the reference for a zero slope, helping to assess whether the trends in PC1 and PC2 for everyone are significant. Additionally, the figure also shows the random interceptions estimates and their confidence intervals for PC1 and PC2, with red dots representing the random interceptions for each and red error bars showing the 95% confidence intervals. The black dashed line for zero intercept serves to reveal whether there are significant differences in the initial values of PC1 and PC2 across individuals. (b) Each curve represents the trajectory of an individual, showing how their PC1 and PC2 scores change at different time points. The curves are smoothed using local weighted regression, and the shaded area around the curve indicates the 95% confidence interval for the trend.

To further investigate interindividual differences in baseline levels and temporal trajectories of spatial behavior, we conducted likelihood ratio tests comparing random intercept‐only models to models including both random intercepts and random slopes. For E×E, inclusion of random slopes significantly improved model fit (*χ* ^2^ = 9.25, df = 2, *p* < 0.05). Similarly, the model comparison for T×C showed a substantial improvement in fit when random slopes were included (*χ* ^2^ = 32.73, df = 2, *p* < 0.05). Random‐intercept and random‐slope are shown in Figure 4.

## 4 Discussion

The balance between behavioral consistency and plasticity shapes how animals adjust to novel environments, but the factors influencing this balance remain poorly understood (Dingemanse et al. 2010; Duckworth 2010). By tracking reintroduced giant pandas in a novel environment, we found detectable and repeatable among‐individual differences in movement, but this variation weakened over time during the early post‐release period. This pattern is consistent with the biology of giant pandas as dietary specialists with low reported energy expenditure (Nie et al. 2015). At the same time, giant panda movement and habitat use also respond to habitat structure and bamboo distribution, and post‐release trajectories may additionally reflect disturbance, learning, and behavioral flexibility (Li et al. 2026; Wei et al. 2015; Zhao et al. 2017). These findings also motivate the conceptual hypothesis developed below.

### 4.1 Among‐Individual Differences and Short‐Term Adjustment After Release

Our analysis identified repeatable among‐individual differences in movement during the early post‐release period. By using the GPS‐tracked movement data, we reduced ten movement metrics into two independent axes and identified a primary E×E continuum and a secondary T×C axis. The statistical independence of E×E and T×C suggests that these two axes captured different aspects of spatial behavior during the early post‐release period. E×E mainly described differences in movement extent and local space reuse, whereas T×C reflected differences in path geometry and directional consistency. Movement‐based metrics have increasingly been used to quantify among‐individual behavioral variation in wild animals, particularly when standardized behavioral assays are difficult to conduct (Hertel et al. 2020; Spiegel et al. 2017). Therefore, variation along the E×E axis may be comparable to exploration‐related behavioral variation often discussed in relation to animal personality and responses to novel environments (Dingemanse et al. 2010; Sih et al. 2004). Lower E×E scores were associated with greater daily distances, higher mean speeds, larger daily maximum diameters, greater mean elevation changes, and larger nearest‐neighbor distances. In contrast, higher E×E scores corresponded to higher repeat ratios and revisit rates, reflecting more localized and repetitive space use. Because these axes were derived from GPS‐based movement metrics, we interpret them as movement‐based spatial‐behavior dimensions and use them to describe post‐release spatial adjustment. Repeatability of both behavioral axes declined significantly over time, eventually stabilizing at a low level. This pattern may reflect behavioral convergence during early adjustment, but increased stochasticity, measurement variation, or behavioral flexibility may also contribute. Initially, individuals may rely more on their baseline movement tendencies, which can lead to greater among‐individual variation in an unfamiliar landscape. The random‐slope models further showed that individuals differed not only in their initial E×E and T×C values but also in how these axes changed over time. This pattern suggests that early post‐release adjustment was not uniform across individuals. Some pandas showed stronger temporal shifts in spatial behavior, whereas others maintained relatively stable movement patterns. Together with the decline in repeatability, these results indicate that the contribution of consistent individual variation to overall movement variability decreased as the early post‐release period progressed.

This temporal pattern may be reflected by several non‐mutually exclusive processes. Among‐individual variance may first undergo contraction. When released into unfamiliar landscapes, animals generally transition gradually from exploration to exploitative behaviors, and this shift often proceeds at different rates and with distinct dynamics across individuals (Heidinger et al. 2009; Picardi et al. 2021). As more individuals adopt increasingly constrained space‐use strategies over time, among‐individual differences along the E×E axis are likely to narrow. A second contributing factor could be an increase in within‐individual variance. Short‐term acclimation entails the accumulation of local experience and habituation to novel environments. Within individuals, daily movement patterns may alternate between exploratory and localized use, which elevates day‐to‐day within‐individual variation and, in turn, lowers the repeatability of both E×E and T×C (Nakagawa and Schielzeth 2010; Wilson 2018). Residual variance may also contribute to the observed pattern. For metrics tied to path geometry, GPS positioning error is prone to introducing spurious 180° turning angles and directional biases (Hurford 2009). Metrics characterizing turning angles and directional persistence are thus particularly vulnerable to such location errors, meaning that the observed decline in T×C may stem from observational noise.

### 4.2 Energetic Limitation as One Possible Explanation

The reduction in variation among individual movement patterns observed here is consistent with the giant panda's low‐energy life‐history traits. In unfamiliar environments, broad exploration may help individuals locate bamboo resources, resting sites, and other key habitat features, but such activity is also likely to entail higher movement costs, including greater travel distances and elevation changes. Because giant pandas subsist on low‐nutrient bamboo and have exceptionally low reported daily energy expenditure (Nie et al. 2015), sustaining prolonged exploratory movement may be difficult. As individuals gain experience in a new area, those that initially explored more widely may gradually reduce their movement range, which may contribute to the narrowing of movement differences among individuals during the early post‐release period. Across the first week after release, individuals with higher exploration scores reduced their movement extent more strongly than those with lower exploration scores, a pattern consistent with energetic limitation.

Wang et al. (2023) reported that wild giant pandas followed individual‐specific, multiphasic movement paths during seasonal migration, suggesting that giant panda movement shows structured individual variation rather than a fully stereotyped spatial pattern. In our study, reintroduced giant pandas also showed detectable among‐individual differences in movement and space use during the early post‐release period (Figure 4a). Individuals with lower E×E scores tended to travel longer distances and make use of a wider spatial range. In contrast, individuals with higher E×E scores showed more concentrated activity, revisited the same area more frequently, and displayed higher local use intensity. During the early post‐release period, individuals with higher E×E scores gradually shifted toward more localized space use (Figure 4b). Energetic limitation provides one plausible explanation for this shift in movement pattern. In the early stages of adapting to a new environment, broader exploration may help pandas find ample space and locate resources, but it may also impose higher movement costs (Hull et al. 2015). Therefore, individuals that initially showed broader exploratory movement may reduce exploration intensity over time, which would weaken among‐individual differences in movement.

Although giant pandas have consumed bamboo for millions of years, their digestive systems retain many carnivore‐like features, and their gut microbiota have limited capacity to degrade cellulose and lignin, resulting in low energy digestibility from bamboo (Finley et al. 2011; Zhang et al. 2018). Consequently, the net energy pandas obtain from bamboo is inherently low. Different bamboo parts also vary markedly in nutritional composition and bioavailability. When pandas feed on high‐fiber culms or leaves, nutrient absorption efficiency often declines further, bringing individuals closer to a negative energy balance (Nie et al. 2019; Wang et al. 2017). Thus, energy gain is continuously constrained by the quality of bamboo parts and their seasonal changes.

Given this energy constraint, movement costs become increasingly important. Movement expenditure can account for a significant portion of a terrestrial animal's daily energy budget. Path selection and space use typically depend on a trade‐off between resource benefits and movement costs, with larger‐scale exploration generally incurring higher energy investment (Hetem et al. 2025; Klappstein et al. 2022). Studies on dispersal in large terrestrial mammals provide direct evidence: individuals that move extensively have about 22% higher energy expenditure than resident individuals and increase their daily travel distance by approximately 63%, indicating that large‐scale movement itself is energetically costly (Benoit et al. 2020).

During the early stage of translocation, individuals with higher exploration intensity scores may experience a sharper increase in movement costs, as they sample the environment over longer distances, at higher speeds, and across broader spatial ranges. As local environmental information accumulates, the additional benefit of maintaining wide‐ranging exploration is likely to decline, yet energy expenditure continues. Consequently, high‐cost exploration is more readily suppressed, whereas localized space use tends to persist (Benoit et al. 2020). State‐dependent behavior theory suggests that behavioral expression is constrained by an individual's underlying state variables. Physiological attributes such as metabolic rate and locomotor capacity influence activity, exploration, and dispersal behavior. As state constraints intensify, the persistence of high‐cost behavior often declines first, and the upper end of the behavioral distribution tends to shrink (Sih et al. 2015; Wu and Seebacher 2022). Accordingly, individuals that initially showed broader exploratory movement may reduce exploration more rapidly during the early post‐release period, thereby compressing among‐individual variation and contributing to the observed decline in repeatability.

### 4.3 A Conceptual Diet–Energy Utilization Constraint Hypothesis

**FIGURE 5:**
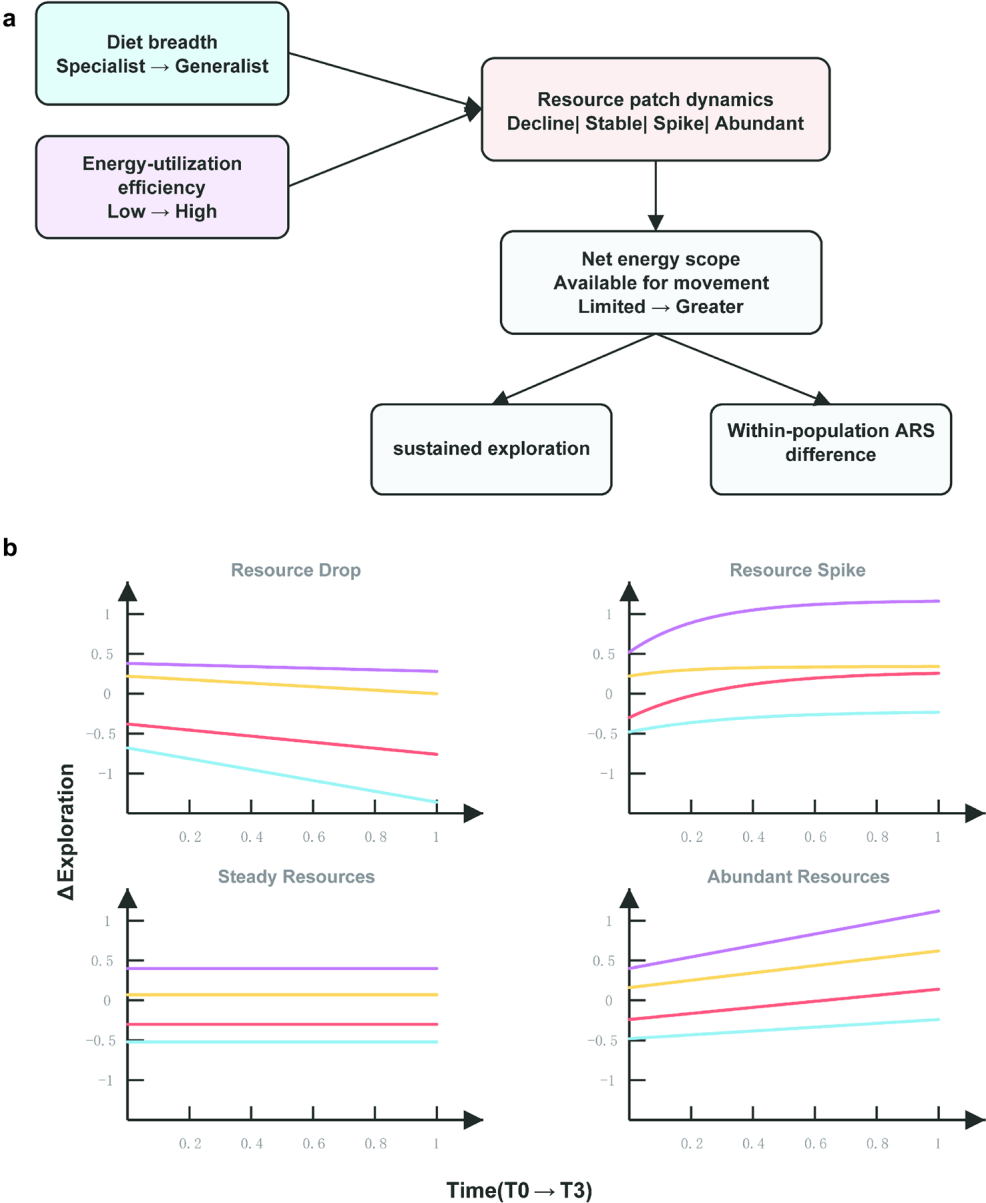
Conceptual schematic of the Diet–Energy Utilization Constraint hypothesis. The upper framework links diet breadth, energy utilization efficiency, and resource‐patch dynamics to net energetic scope, sustained exploration, and within‐population differences in area‐restricted search (ARS). The lower panels show heuristic predictions for temporal changes in within‐population ARS differences under four resource‐patch scenarios: Resource decline, resource increase, stable resources, and abundant resources. The blue, red, yellow, and purple lines represent Specialist‐Low energy efficiency, Specialist‐High energy efficiency, Generalist‐Low energy efficiency, and Generalist‐High energy efficiency species, respectively. Under resource decline, ARS differences are predicted to contract most strongly in Specialist‐Low energy efficiency species and least strongly in Generalist‐High energy efficiency species. Under resource increase, ARS differences are predicted to expand most clearly in Generalist‐High energy efficiency species, whereas Specialist‐Low energy efficiency species remain comparatively constrained. Under stable or abundant resources, broader diets and higher energy utilization efficiency are expected to maintain greater within‐population ARS differences, whereas narrow diets and low energy utilization efficiency are expected to constrain divergence. The curves are conceptual predictions intended to guide future testing.

Our results motivate a conceptual Diet–Energy Utilization Constraint hypothesis about how dietary specialization and energetic scope may influence movement adjustment after release (Figure 5). Spiegel et al. (2017) proposed that as resource patches become more concentrated, foraging responses may shift toward more reactive and localized tactics. This response may depend not only on the interaction between individual tendencies and resource distribution, but also on net energetic return, which reflects trade‐offs among movement cost, metabolic rate, and energy intake (Shepard et al. 2013; Sotillo et al. 2019; Tamburello et al. 2015). Under this conceptual hypothesis, species with broader diets and greater energetic scope may maintain stronger among‐individual variation under resource uncertainty. In contrast, species with narrower diets and more limited energetic scope may show weaker behavioral divergence.

Diet–energy utilization constrained hypothesis we propose provides a potential mechanistic link between life‐history, individual behavior, and species resilience, and may offer a novel view through which to consider extinction risk. In Figure 5, the phase terminology (T0–T3) and Δ‐exploration function as heuristic descriptors of the conceptual sequence and illustrate predicted patterns of change in movement adjustment. This conceptual hypothesis provides several testable predictions for future research. First, when the patch resource declines, the ARS difference would decrease over time, with the strongest contraction in Specialist‐Low energy efficiency species and the weakest contraction in Generalist‐High energy efficiency species. Second, when patch resources increase, ARS differences would widen most clearly in Generalist‐High energy efficiency species, whereas Specialist‐Low energy efficiency species would remain comparatively constrained. Third, when patch resources remain stable or abundant, Generalist‐High energy efficiency species would maintain the largest ARS differences, whereas Specialist‐Low energy efficiency species would retain the lowest level of divergence.

### 4.4 Implications for Reintroduction and Future Dispersal Research

The implications of this constrained adaptability are profound, particularly for conservation. The slow, multi‐week adaptation period we observed may help explain why reintroduction programs for giant pandas can have high failure rates (Wei 2022). The deaths of “Xiangxiang” and “XueXue” during this adjustment window further highlight the vulnerability of individuals before they establish stable space‐use patterns in unfamiliar environments. These findings have important implications for reintroduction practice. The gradual, multi‐week adjustment period observed in this study may help explain why the initial weeks after release are a particularly vulnerable stage for reintroduced giant pandas, before they establish stable space‐use patterns in unfamiliar environments. While our study did not directly compare release protocols, our results suggest that management strategies to reduce early exploration costs merit further evaluation. For instance, soft‐release approaches that span the early adjustment period, such as gradual access to the release site, temporary resource support, or intensified monitoring during the first weeks to 2 months after release, could be contrasted with hard‐release methods that provide no post‐release support. Such comparisons would help determine whether minimizing early movement costs improves settlement and survival outcomes in reintroduced giant pandas.

Early post‐release adjustment, as observed in this study, may also contribute to understanding the search‐and‐settlement phase of dispersal among subadult giant pandas, beyond its relevance to reintroduction efforts. The subadults released here are at an age when wild individuals often begin exploratory movements associated with dispersal. While genetic evidence has suggested female‐biased dispersal in giant pandas, descriptions of early movement patterns based on GPS data remain limited, primarily due to the challenges of tracking this endangered species in the wild (Hu et al. 2010; Zhan et al. 2007). Although release into a new habitat and natural natal dispersal differ in several respects, both situations require subadults to navigate unfamiliar landscapes, locate suitable bamboo, identify available habitat, and gradually establish stable patterns of space use. In this context, the observed shift from broad exploration to more localized activity may serve as a behavioral reference for the initial adjustment period during dispersal. As direct observation of natural dispersal is uncommon, post‐release tracking can provide a valuable perspective on how subadult giant pandas organize their spatial behavior when entering new environments, a process otherwise difficult to document in the wild.

### 4.5 Limitations and Future Directions

Despite our finding of individual differences in movement among giant pandas as a low‐energy limited species after initial release, and decrease of these differences over time, some limitations need to be addressed. First, our sample size is small, and the tracking window captures only early post‐release adjustment. And landscape context may also shape the observed shift (Picardi et al. 2022; Zecherle et al. 2020). In addition, habitat fragmentation and limited connectivity can restrict long‐distance exploration by reducing chances of access to suitable patches and lower‐risk corridors (Amaral et al. 2016; Gilbert‐Norton et al. 2010; Stewart et al. 2019). If connectivity constrains movement, individuals with a higher range of movement may show a stronger reduction in movement distance or exploration, making movement strategies appear more similar over time (Atkins et al. 2019; Robles et al. 2022). Our study did not quantify fragmentation or corridor use, so we could not test this mechanism here. Therefore, future work should combine post‐release tracking with measures of habitat structure, resource distribution, and connectivity across sites in different fragmentation.

In summary, our results show that reintroduced giant pandas display detectable among‐individual differences in movement shortly after release, but these differences weaken during early post‐release adjustment. This pattern highlights the importance of considering both individual variation and potential energetic constraints when interpreting post‐release movement and when designing reintroduction monitoring and site selection.

## Author Contributions

**Jianghong Ran:** conceptualization (lead), data curation (equal), formal analysis (equal), funding acquisition (equal). **Yuxin Jiang:** conceptualization (equal), formal analysis (lead). **Shengshan He:** investigation (equal). **Tianjiao Liu:** formal analysis (equal). **Yuhang Li:** writing – original draft (supporting). **Yuanhao Du:** writing – review and editing (equal). **Xiaoqiang Liu:** investigation (lead). **Qian Wang:** investigation (supporting). **Haohong Wan:** investigation (supporting). **Desheng Li:** funding acquisition (lead).

## Funding

This work was supported by the project “Study on Key Technologies for Conservation of Wild Giant Panda Populations and Its Habitats within Giant Panda National Park System” (China Green Foundation, Grant No. CGF2024001).

## Ethics Statement

No animal experiments were conducted as part of this study. All behavioral and movement data were obtained from previously approved and archived monitoring records under the authority of the China Conservation and Research Center for the Giant Panda (CCRCGP).

## Conflicts of Interest

The authors declare no conflicts of interest.

## Supporting information


**Figure S1:** Temporal dynamics of ten movement metrics across individuals. Solid colored lines and points represent daily values for each individual; Panels (a)–(j) correspond, respectively, to daily total distance, mean turning angle, straightness index, mean speed, repeat ratio, maximum path diameter, revisit rate, direction consistency, mean elevation change, and mean nearest‑neighbor distance.
**Table S1:** Full PCA loadings of the ten daily movement metrics on PC1 and PC2.

## Data Availability

All the required data are uploaded as Supporting Information.
