# Supplementary material for: Early Post‐Release Movement Convergence in Reintroduced Giant Pandas
Source: Ecol Evol. 2026 Jun 9;16(6):e73807. doi: 10.1002/ece3.73807 (PMC13247555; doi:10.1002/ece3.73807)
Supplement: Supplementary file 1 — Figure S1: Temporal dynamics of ten movement metrics across individuals. Solid colored lines and points represent daily values for each individual; Panels (a)–(j) correspond, respectively, to daily total distance, mean turning angle, straightness index, mean speed, repeat ratio, maximum path diameter, revisit rate, direction consistency, mean elevation change, and mean nearest‑neighbor distance. Table S1: Full PCA loadings of the ten daily movement metrics on PC1 and PC2. [file ECE3-16-e73807-s001.docx]

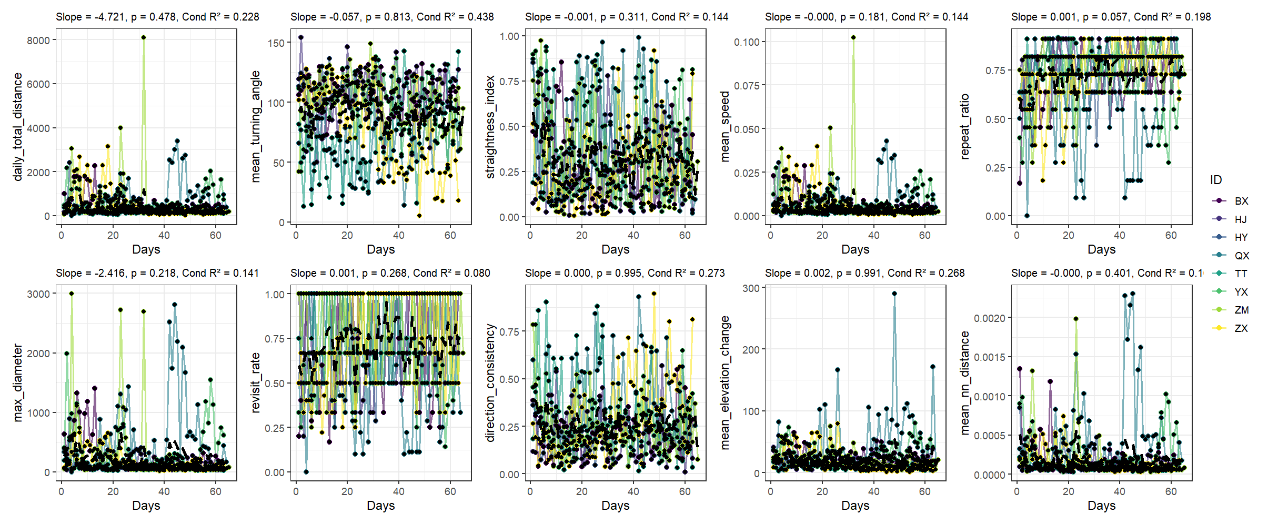


**Figure S1.**Temporal dynamics of ten movement metrics across individuals. Solid colored lines and points represent daily values for each individual; Panels (a)–(j) correspond, respectively, to daily total distance, mean turning angle, straightness index, mean speed, repeat ratio, maximum path diameter, revisit rate, direction consistency, mean elevation change, and mean nearest‑neighbour distance.

| Table S1. Full PCA loadings of the ten daily movement metrics on PC1and PC2 | | |
| --- | --- | --- |
| Movement metric | PC1 loading | PC2 loading |
| Daily total distance | **-0.386** | 0.194 |
| Mean turning angle | 0.074 | **0.594** |
| Straightness index | -0.205 | **-0.42** |
| Mean speed | **-0.37** | 0.132 |
| Repeat ratio | **0.388** | 0.028 |
| Max diameter | **-0.406** | 0.015 |
| Revisit rate | **0.313** | -0.067 |
| Direction consistency | -0.126 | **-0.576** |
| Mean elevation change | **-0.30** | 0.276 |
| Mean nearest neighboring distance | **-0.383** | -0.054 |
| **Note.** Loadings with an absolute value ≥ 0.30 were used to aid ecological interpretation of the two axes. | | |
